# New Facile Testing Protocols to Assess Conformability of Advanced Dressings for Wound Healing

**DOI:** 10.3390/ijms27188149

**Published:** 2026-09-13

**Authors:** Daniele Ghezzi, Gabriela Graziani

**Affiliations:** 1 Department of Pharmacy and Biotechnology, University of Bologna, 40126 Bologna, Italy; daniele.ghezzi@unibo.it; 2 Human Sciences and Life Quality Promotion Department, San Raffaele Rome University, 00166 Rome, Italy

**Keywords:** wound dressings, exudate, conformability, skin model, skin adaptability

## Abstract

Conformability of advanced dressings is a key parameter to evaluate their ability to adapt to the complex shape of the wound, which promotes its healing and prevents the formation of gaps at the wound interface, which, in turn, may favour microbial proliferation. However, conformability is normally measured by evaluating the mechanical properties of dressings and frequently under dry conditions, failing to evaluate the behaviour in clinical conditions, where the wound is normally characterized by large volumes of exudate. New methods are under study for its evaluation, but they generally require complex experimental procedures or specialized equipment. Here, we validate a simple and inexpensive method proposed for conformability characterization by testing its application to a variety of dressings, differing in composition and type of absorbing layer. We test the impact of different volumes of exudate-simulating solution and the impact of dressing size on the results. In addition, we implement the test by simultaneously evaluating other parameters (time of absorption, lateral spreading of the solution) that impact wound healing. Finally, we validate the test by demonstrating its capability to predict adaptability of complex wound shapes in specifically developed wound-mimicking models. Our results show that the test can be applied to all tested dressings, successfully highlighting differences in conformability and demonstrating high reproducibility. We demonstrate that conformability can largely vary between different dressings but is not affected by either dressing size or exudate volume, whereas lateral spreading and time of absorption can be significantly affected by both parameters. Finally, we demonstrate that the tests succeed in predicting adaptability to wounds of complex shape in tissue models; hence it is suitable for a simple and cost-effective measure of conformability.

## 1. Introduction

Conformability, i.e., the ability to adapt to irregular, deep, or high-mobility wound contours, is among the key requirements of advanced dressings, as it ensures consistent contact with the wound bed and effective management of high volumes of exudates [1,2,3]. By deforming and adapting to the wound shape, a conformable dressing can prevent the formation of empty space between the dressing and the wound, which is crucial for ensuring patient comfort, prolonged adhesion and, most importantly, reducing the risk of infection and promoting tissue healing [4].

Indeed, dermal wounds are highly susceptible to bacterial colonization and infection. When infection occurs, it can be difficult to eradicate and interferes with tissue regeneration [5,6]. Healing, instead, can be promoted by contact with the dressing, exploiting morphological cues, while inefficient adaptation to the wound complex shape can cause mechanical stress, leading to hampered tissue healing [6,7,8,9].

As a consequence, conformability of dressings is one of the key factors that can lead to insufficient performance, so its evaluation is of high importance [10].

Conformability can be measured following the standard BS EN 13726-4:2003 and 2023 [11], which aims to evaluate “aspects of conformability” based on extensibility and permanent set under tensile strength. However, the standard only refers to adaptability to the anatomical site and not to the lesion. In addition, there is no general agreement on this parameter, as it fails to evaluate the ability of the dressing to change its shape, adapting to the complex 3D contour of a wound [12,13]. In addition, the test is conducted on dry, conditioned samples at standardized humidity and temperature. While this improves reproducibility, current wound dressings in clinical use are often wet [14]. Moisture can significantly change the mechanical properties of the dressing and exudate absorption can cause swelling, which modifies geometry and, in turn, also impacts mechanical properties [15]. Most importantly, some dressings can jellify upon wetting, which results in a completely different mechanical behaviour [16].

For this reason, a multitude of tests has been employed to measure conformability, including simple tests, such as drape and bending test, or more complex ones, such as dynamic joint or articulated limb tests, soft tissue models, pressure mapping or contact area analysis [12,16,17,18,19]. However, drape and bending also suffer issues connected to exudate draining. Indeed, although samples can be prewetted, this does not reproduce the modifications caused by draining exudate (which are local) and does not provide information about exudate draining/spreading [19]. As for the other tests, they require complex and costly testing apparatuses and/or long procedures.

Recently, Brennan et al. [20], proposed the proof of concept of a new protocol for validation of conformability of advanced dressings, based on Relative Swelling Rise (RSR). However, being recently introduced, the paper only tested the conformability of one type of dressing, without validating its applicability to different dressing types and compositions, and hence its capability to discriminate between dressings having different conformability and/or to recognize and distinguish high and low conformability. In addition, the test has not yet been validated in comparison with different protocols to demonstrate its ability to predict adaptability to complex wound shapes.

For this reason, here, we investigate the appropriateness of this method for evaluating conformability, dressing behaviour, and the ability of dressings to adapt their shape upon contact with the exudate. To this end, we compare a variety of dressings differing in absorbing layer material, type, and size and assess the applicability of the test and its ability to detect differences in conformability among the different dressings. Through this comparison, we also aim at evaluating whether conformability varies significantly among different dressings and whether it correlates with inadequate exudate drainage. To the latter aim, we include the evaluation of absorption time and lateral spreading of the exudate as key parameters to be evaluated during the test.

Finally, we validate the model and demonstrate its ability to assess adaptation to complex wound shapes and absorption of high volumes of exudate, under conditions simulating a real-world scenario. To this aim, we test the behaviour of the dressings showing high conformability by a specifically developed wound model. This model consists of a mould with a complex shape, a gelatin layer simulating the skin, and a liquid layer simulating exudate.

Together with validating the test, we exploit it to drive considerations about the correlation between conformability and dressing composition and technology, with the testing parameters (amount of solution, dressing size) and with the ability to correctly absorb the exudate.

## 2. Results and Discussion

### 2.1. Amount of Absorbed Solution and Absorption Time

Data regarding the amount of absorbed solution are shown in Table 1.

Data show that all the dressings absorb the whole amount of solution, with no significant contributions from evaporation or accidental losses, resulting in β values, i.e., the ratio between the increase in dressing weight and the amount of added solution, ranging from 0.96 to 1 for all dressings (for calculation of beta see the Methods Section 3). The ability of the dressings to completely absorb the test solution within 30 min does not significantly depend on either the size of the dressing or that of the fence (i.e., on the volume of solution), and no relevant differences are assessed between different materials. This indicates that all the dressings show high efficacy in draining the exudate and that this is not a sensitive parameter to distinguish between them.

**Figure 1:**
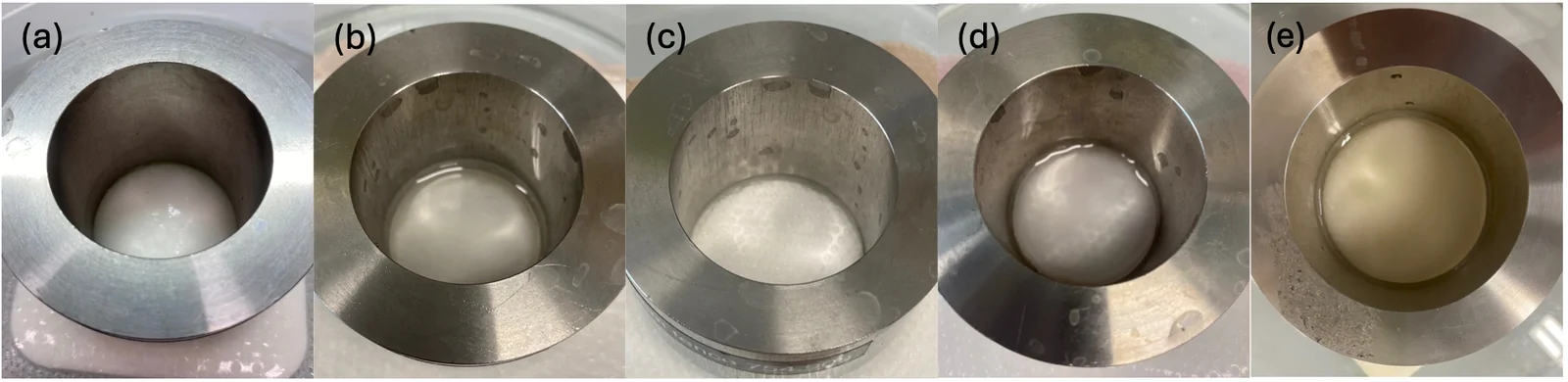
Different dressings show different absorption at time of 2 min. (**a**) Silicon foam; (**b**) carboxymethylated spun cellulose fibres-based dressings; (**c**) polyurethane foam; (**d**) hydrocellular polyurethane foam; (**e**) hydrocellular lipido-colloid dressing. Delayed absorption is observed in (**b**,**d**,**e**).

However, significant differences are observed in the time needed to absorb the exudate-simulating solution (Figure 1).

More specifically, for the silicon foam samples, absorption is almost instantaneous in all dressings, particularly for the larger ones, with a maximum time of about 1.5 min. Then, absorption follows steadily if the liquid keeps being poured. In this case, the absorption time decreases as the size of the dressing increases and does not increase with the diameter of the fence (and therefore the volume of solution in contact with it).

Similarly, the absorption time is also instantaneous for the polyurethane foam dressings.

For the carboxymethylated spun cellulose fibres-based dressings (the latter measured only in the 15 × 15 size), absorption is slower (minimum time between 2 and 4 min) and increases significantly with increasing dressing size and fence diameter, exceeding 5 min for the combination of size 15 and fence 60 (Table 1).

Absorption times of the same order of magnitude (2–4 min for smaller dressings and up to 6 min) are also recorded for the hydrocellular polyurethane foam and hydrocellular lipido-colloid dressings (Table 1).

For these latter three dressings, the relatively long absorption time, which increases with the solution volume, may indicate reduced drainage capacity for wounds with high exudate.

### 2.2. Swelling: Macroscopic Observations

**Figure 2:**
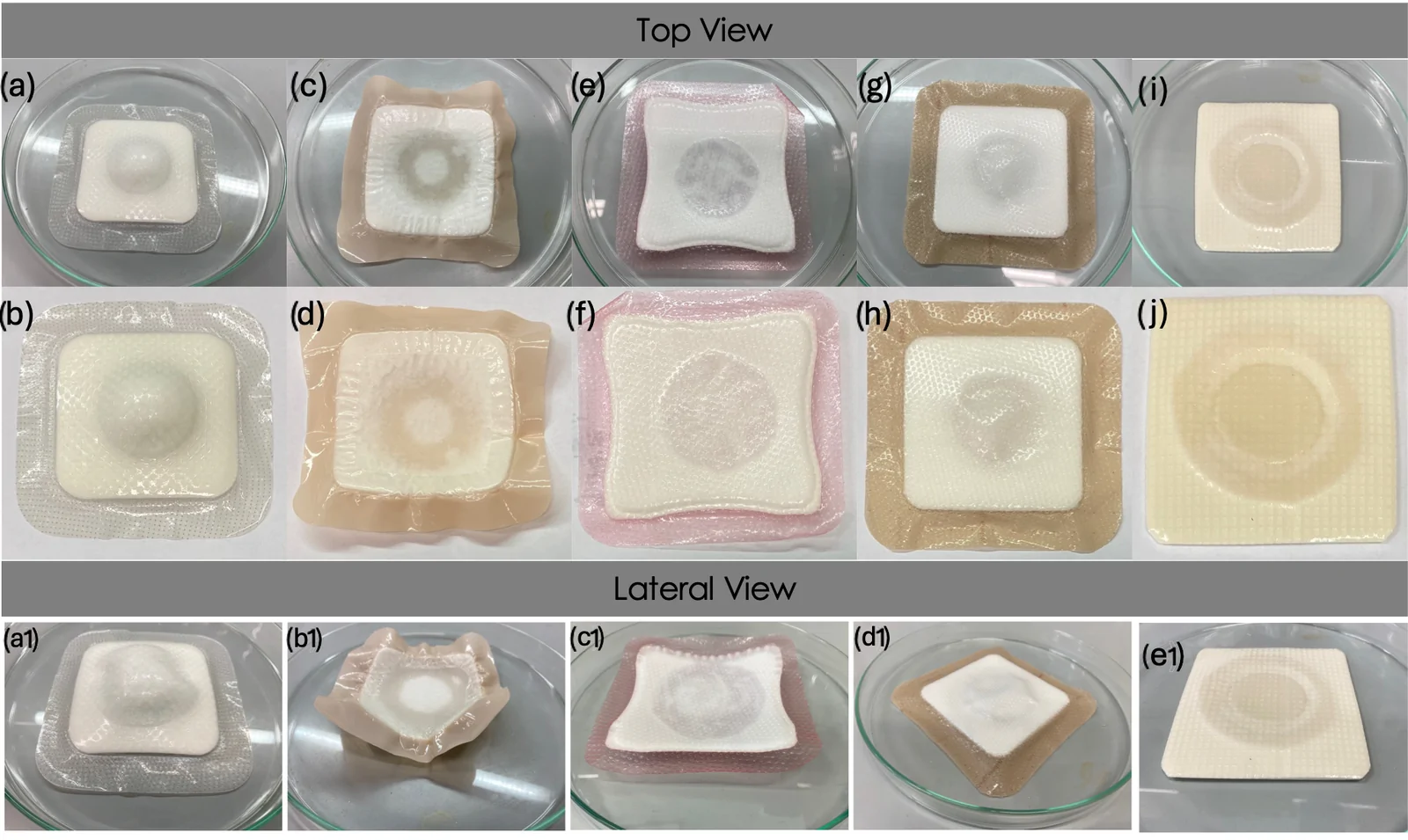
Behaviour of the dressings in contact with the simulated exudate. Top view: (**a**,**b**) Silicon foam; (**c**,**d**) carboxymethylated spun cellulose fibres-based dressings; (**e**,**f**) hydrocellular polyurethane foam; (**g**,**h**) polyurethane foam; (**i**,**j**) hydrocellular lipido-colloid dressing. Lateral view: (**a1**) Silicon foam; (**b1**) carboxymethylated spun cellulose fibres-based dressings; (**c1**) hydrocellular polyurethane foam; (**d1**) polyurethane foam; (**e1**) hydrocellular lipido-colloid dressing.

From macroscopic observations related to the absorption of the test solution by the samples (Figure 2), the following considerations can be drawn.

**Figure 3:**
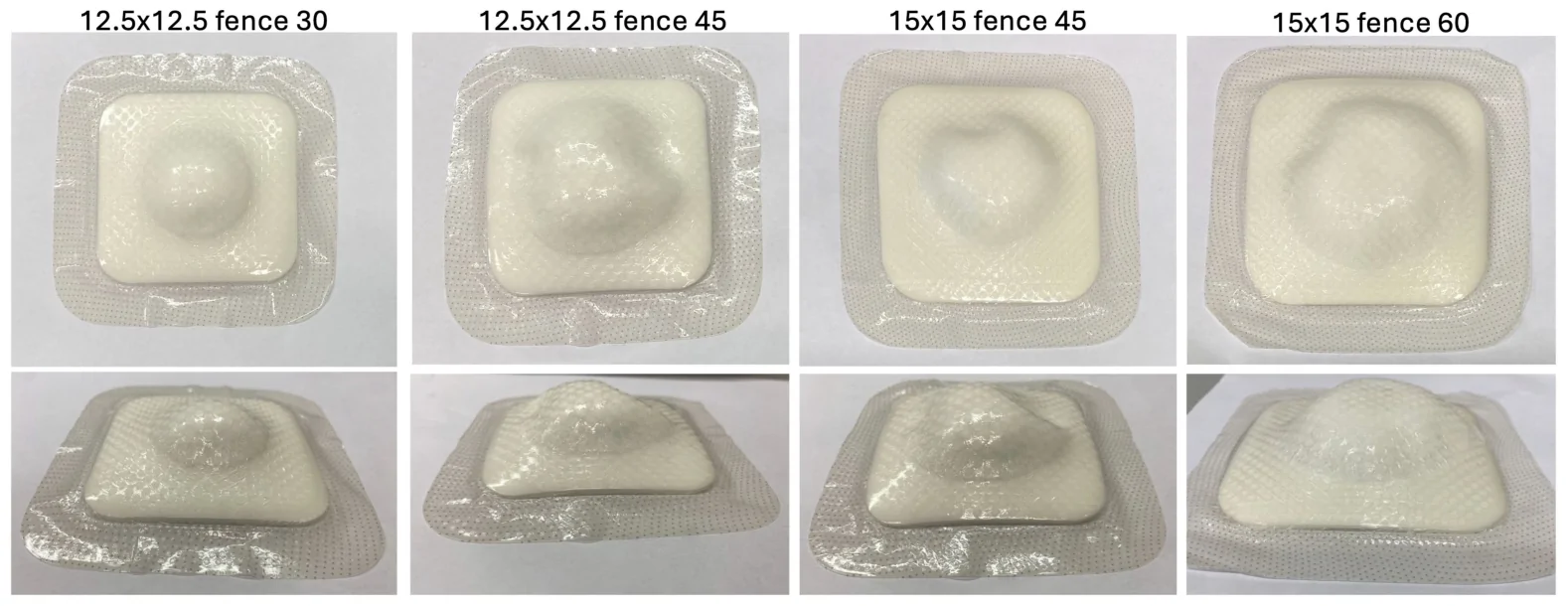
For silicon foam dressings, the behaviour is negligent of the dressing size and the exudate volume.

For the silicon foam samples, a bubble forms already during the liquid absorption phase (Figure 3). The test solution remains confined within the bubble area, and no visible changes in thickness or morphology are observed in the surrounding areas. This characteristic confirms adaptability to the wound bed. To the touch, the dressing appears dry. The behaviour of the samples is independent of dressing type and size and fence diameter (Figure 3).

**Figure 4:**
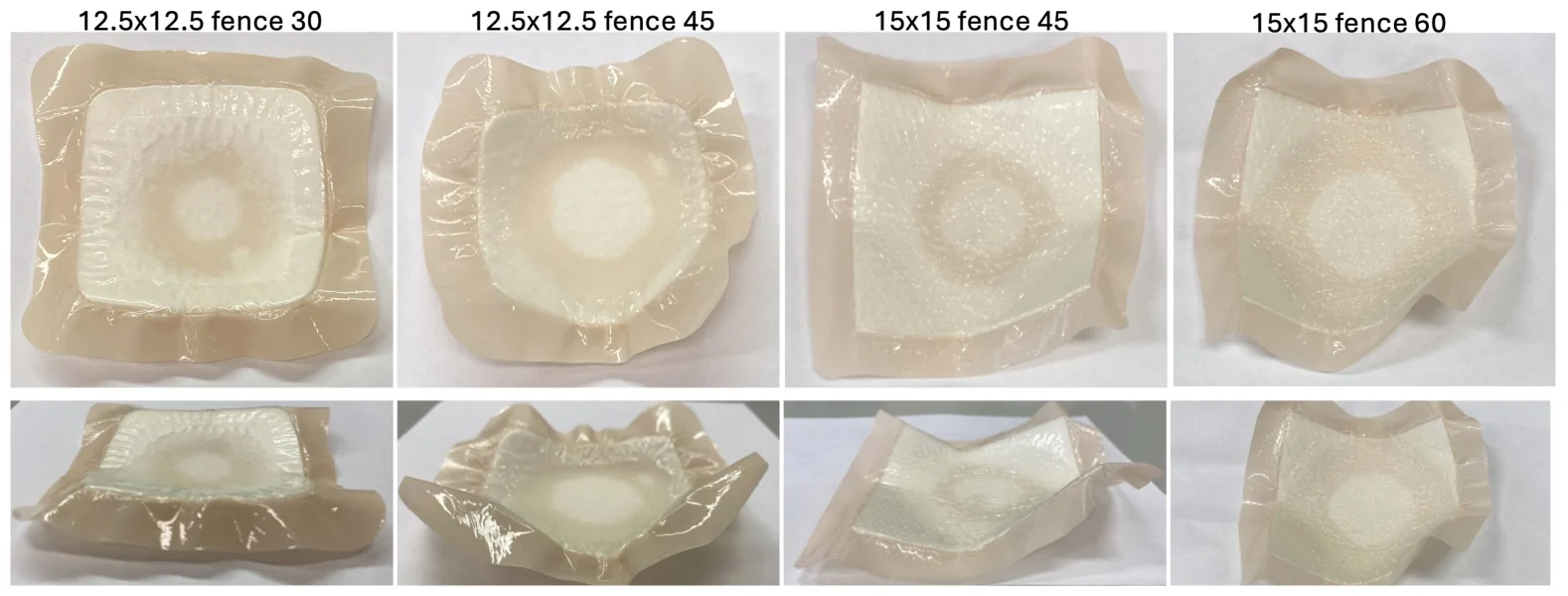
Carboxymethylated spun cellulose fibres-based samples. The behaviour is negligent of the dressing size and the exudate volume.

For carboxymethylated spun cellulose fibres-based samples, the test solution spreads throughout the entire dressing (Figure 4). At the same time, the dressing tends to bend, changing shape and leading to the formation of folds, without evident thickness variations, especially for the 12.5 × 12.5 dressings. This indicates that the dressing has a high capacity for deformation, which permits adhesion to the wound, at least for moderate complexity in its shape. No significant differences in the dressing behaviour are assessed for different size and/or exudate volumes (Figure 4). To the touch, the dressing feels wet.

Hydrocellular polyurethane foam samples show spreading of the test solution beyond the fence area for the 12.5 × 12.5 dressing, although less pronounced than in the cellulose case (Figure 2). To the touch, the dressing feels wet. The 15 × 15 dressing, on the other hand, shows no spreading and feels dry.

For polyurethane foam dressings, no bubble formation or visible change in dressing thickness is observed (Figure 2). The solution appears to remain confined within the fence area. In this region, a swelling is observed, limited to the outer film of the dressing and remaining below the minimum conformability threshold.

The hydrocellular lipido-colloid dressing shows spreading of the test solution beyond the fence area, which was significantly dependent on the fence diameter and became more extensive with increasing volumes of test solution (Figure 2). To the touch, the dressing feels dry. Visually, a lifting of the upper layer of the outer film is evident; however, this does not correspond to bubble formation, as it is not accompanied by swelling of the underlying absorbent layer (Supplementary Materials, Figure S1).

These results indicate that swelling mainly correlates with the composition of the dressing, with foams performing better in terms of adaptability. Instead, the behaviour strongly correlates with the type of polymer: synthetic polymer foams better limit lateral spread, whereas hydrocolloids (natural polymers) and hydrocellular polyurethane foams are characterized by extensive spreading.

The spreading of the solution beyond the fence shall be considered a negative aspect, as it means the exudate may spread beyond the affected area and onto healthy skin. In addition, particular attention shall be paid to dressings whose surface remains wet. Indeed, excessive moisture in contact with the damaged tissue can lead to maceration of the wound bed and the surrounding healthy tissue. Therefore, the ability to absorb excess exudates from the wound bed is preferable [5].

Interestingly, the test is able to identify the different behaviour of the dressings already visually, distinguishing between different degrees of conformability: no modification, shape adaptability, and bubble formation.

Finally, data show that conformability does not correlate with lateral spreading or with the ability to maintain a dry surface. Therefore, all parameters shall be evaluated separately, although the proposed test allows their simultaneous assessment.

The visual observations are confirmed by quantitative data, which are reported in Table 2.

Bubble formation within the fences is observed only for the silicon foam samples. Accordingly, the height after solution absorption and the average height increase (hnet, μ average) are significantly higher than the threshold values and exceed by one order of magnitude the values reached by all other dressings.

For the latter, all alpha values, i.e., the ratios between the average net bubble height and the barrier diameter, are below the limit value of 0.2, indicated as significant for bubble formation, and all bubble heights (hnet,μ average) values are below 1.4 cm.

For the silicon foam dressings, a dependence of alpha on dressing size is observed (alpha is higher for smaller dressings, even at the same fence diameter), while no significant dependence on fence diameter is observed for a given dressing. The alpha values observed in this experiment are consistent with those reported in a previous study [20]. Specifically, values of 0.47 and 0.46 are observed for the two fences (compared to 0.48 in the publication) for the 12.5 × 12.5 dressings and 0.33 and 0.39 for the two fences (compared to 0.36 in the publication) for the 15 × 15 dressings, confirming the reproducibility of the test. Examining the bubble height, it is observed that for all dressing sizes and fence diameters, this value exceeds 1.4 cm, defined as the minimum threshold for dressing RSR. For a given dressing size, the height increases with increasing fence diameter (consistent with the greater absorbed solution, β) and with increasing dressing size. For a given fence diameter, the height is lower for the larger dressing.

**Figure 5:**
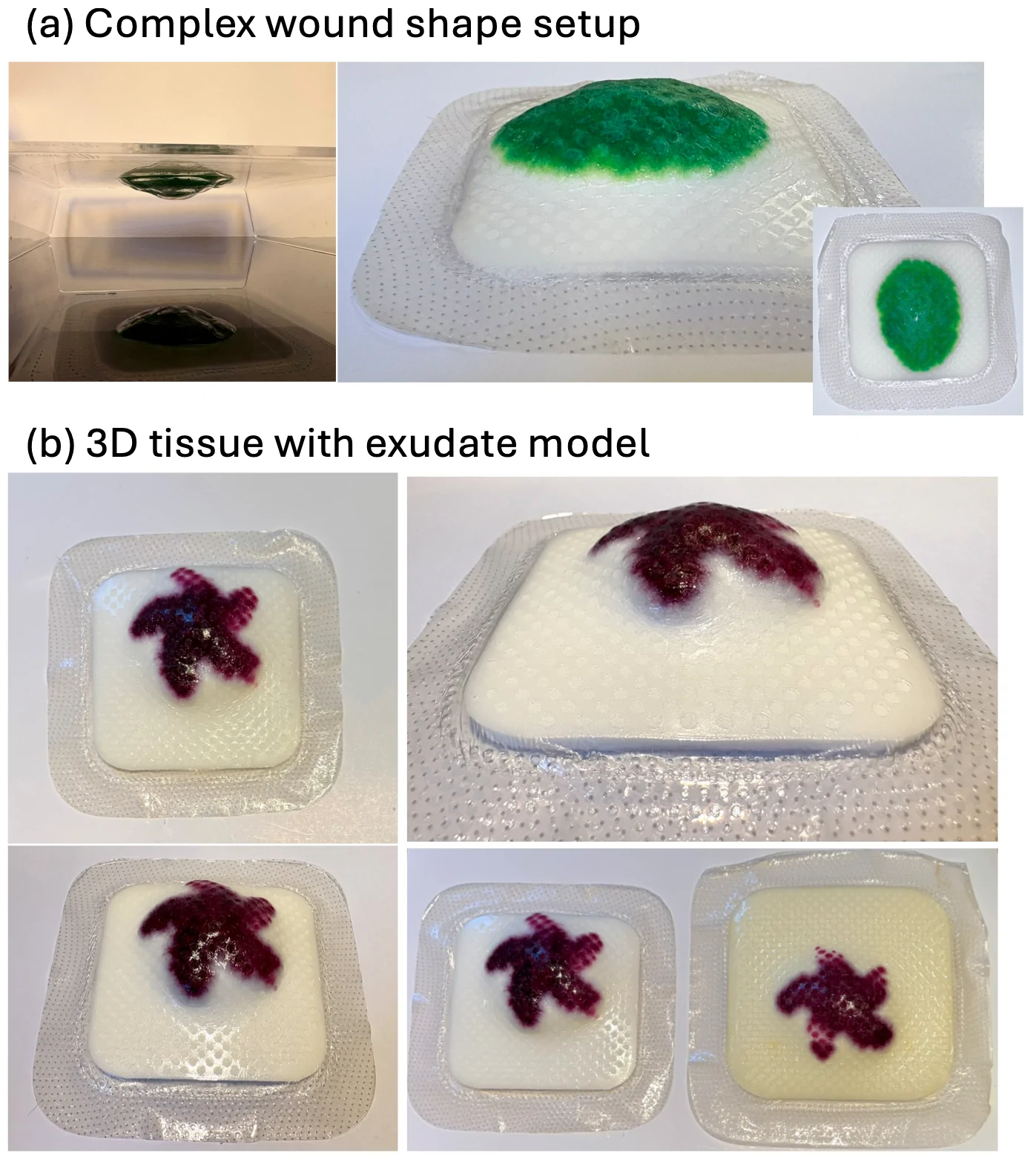
Conformability is verified by (**a**) a complex shape mould and (**b**) a tissue-mimicking mould.

When put in contact with the complex shape mould (Figure 5a), the high-conformability dressing adapts to the different heights and to the valleys, highlighting the ability of the bubble method to predict conformability.

In the case of the use of the simulated tissue, we observe that the absorption of fluid is not delayed by the presence of the tissue-mimicking hydrogel (Figure 5b). The conformability is again testified by the fact that the medication adapts to the complex shape, and no spreading of the solution is noticed in any area not directly in contact with it.

## 3. Materials and Methods

### 3.1. Materials

For the tests, a simulated exudate solution was prepared, as defined in ISO 13726-1:2002 [21]. For its preparation, we used:

- Calcium chloride di-hydrate (CaCl_2_.2H_2_O), Sigma Aldrich (St. Louis, MO, USA);
- Sodium chloride (NaCl), Sigma Aldrich.

Tests were performed on a commercial, high-conformability dressing in silicon foam (Biatain Silicone, Coloplast A/S, Humlebæk, Denmark) and compared with dressings of the same geometry (thickness of the absorbing layer) but different materials:

- Carboxymethylated spun cellulose fibres-based dressings
- Hydrocellular polyurethane foam
- Polyurethane foam
- Hydrocellular lipido-colloid dressing

All dressings have been tested in the 12 × 12 cm^2^ size. To investigate the effect of the adsorbing surface size on conformability, one high- and one medium-conformability material were also tested on a larger size, i.e., 15 × 15 cm^2^.

Dressings have been tested against two different volumes of liquid by using metallic fences of different diameter: 3 and 4.5 cm for dressings of size 12 × 12 cm^2^, 45 and 6 cm for sizes 15 × 15 cm^2^. Metallic fences permit the liquid to remain confined in a determined region, thus simulating the wound while maintaining a constant pressure.

### 3.2. Testing Protocols

#### 3.2.1. Amount of Absorbed Solution and Absorption Time

Following ISO 13726-1:2002 [21], a solution mimicking human exudate was prepared using 142 mM sodium chloride and 2.5 mM calcium chloride in deionized water.

All samples were kept at ambient conditions until testing. The free swell absorptive capacity of the dressings was tested by immersing the entire specimens in the solution warmed at (37 ± 1) °C and kept in a ventilated oven at 37 °C for 30 min.

Based on the weight of the absorbed solution, the absorption capacity was measured (Φ100%), defined as the average mass retained by 100 cm^2^ dressing surface. For all the further tests, we used an amount of liquid corresponding to 80% of Φ100% and defined by the formula:

$$Volume=fence\;area\times \frac{80\%}{100\%}\times \Phi 100\%$$

Height and dry weight were measured for all dressings. Height was measured at 3 random points and in 2 perpendicular directions using a calliper.

Then, the dressings were put in contact with the test solution by pipetting (Supplementary Materials, Figure S2).

Two diameters of fences were used for each size of dressing to study reproducibility and possible dependence of conformability on the volume of test solution. Absorption time was measured starting from the instant of contact between the dressing and the test solution. The test solution was left in contact with the dressing until complete absorption, which was visually measured; then total time for absorption was recorded.

Following complete absorption, the final weight and height of the patch were measured, again using the calliper.

To evaluate the amount of solution absorbed, the final (wet) weight of each sample is measured and compared with the previously recorded dry weight. The difference in weight, calculated according to the formula Wet weight [g] − Dry weight [g] provides the amount of absorbed fluid. This value serves as a control, as it allows comparison between the amount of test solution applied to the dressing and the amount actually absorbed, taking into account evaporation, any losses during pipetting, or other significant deviations. This enables the controlled calculation of the volume β:

$$\beta =\frac{Wet\;weight-Dry\;weight}{Added\;fluid\;weight}\times \Phi 100$$

#### 3.2.2. Free Swelling Rise

While measuring swelling, the appearance of the surface and the lateral spreading of the solutions (outside of the fence) were visually evaluated as measures of the capability to maintain a dry environment and the wound interface and to avoid spreading of the exudate in the healthy tissue, respectively.

Swelling was first measured quantitatively by observing formation of a bubble inside the fence and/or significant increase in thickness.

Then, the increase in thickness was evaluated by averaging three measures at 3 random points and two perpendicular directions. The net increase in thickness was defined as hnet = hgross − hdry.

Then, based on this value, we measured the Relative Swelling Rise (RSR):

$$\alpha =\frac{hnet,\mu }{d}=\frac{hgross,\mu -hdry}{d}$$

where

• alpha (α) is the ratio between the average net bubble height and the barrier diameter (measured in mm);
• hdry is the dry thickness of the dressing (measured in mm);
• hnet and hgross are the net height and the gross height of the dressing bubble, respectively (measured in mm).

### 3.3. Validation

The method was validated by testing its capability to predict the adaptability of the high-conformability dressing to complex wound shapes, simulating irregular shapes and height profiles.

To do so, first, we used a mould with a complex height profile, involving a profile with different heights in different parts as well as the presence of valleys.

**Figure 6:**
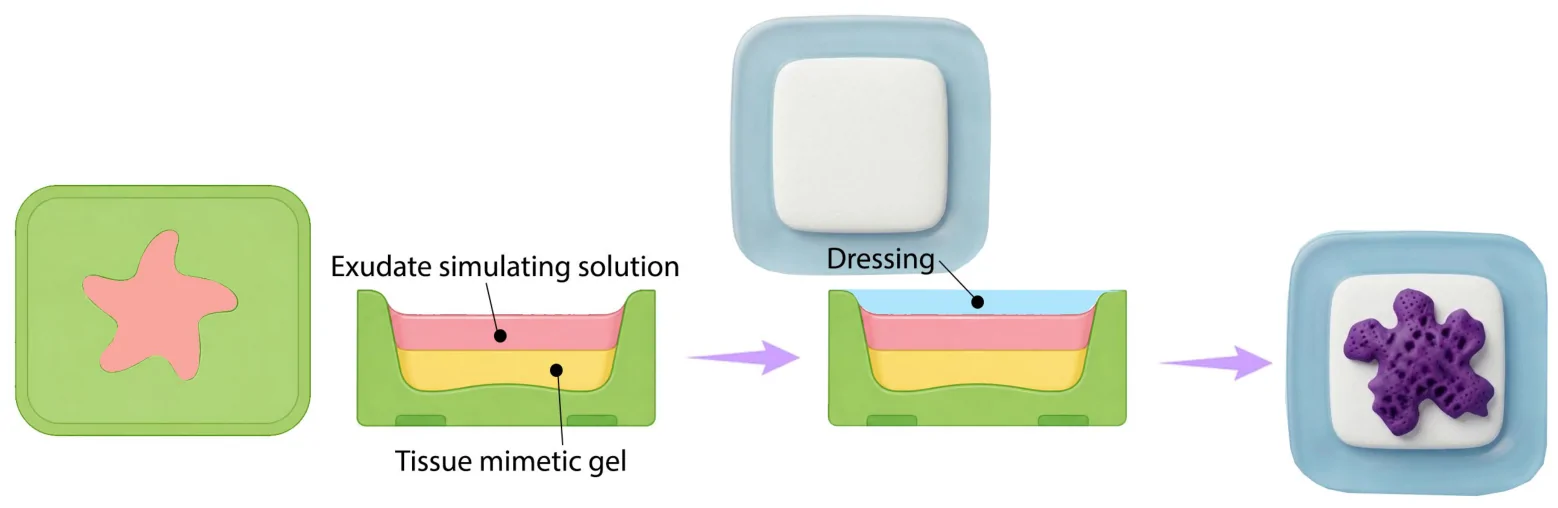
Tissue and exudate-mimicking test.

Then, we developed a simplified 3D tissue model to evaluate the conformability of the dressing in a relevant wound environment (Figure 6). To do so, we used a mould that was filled with a tissue-mimicking layer (10 mm thickness) and an overlapping exudate-simulating layer (5 mm). The tissue-mimicking layer was obtained using gelatine type B (Sigma Aldrich), 10% *w*/*v*, crosslinked with microbial transglutaminase (mtg, TGzyme, Handary S.A., Brussels, Belgium, 20 U/g). The exudate-mimicking liquid was obtained as in Section 3.1. An edible food stain was used to make the visualization of the absorbed solution easier.

The dressing was put in contact with the complex mould and with the tissue model with the adsorbing side in contact with the fluid for 5 min (based on the previously measured absorption time); then the medications were examined to assess their capability to adapt to the complex shapes.

## 4. Conclusions

The data obtained validate the proposed method by demonstrating its applicability to different types of dressings and its ability to discriminate between high and low conformability and to predict the ability to adapt to the wound bed, including for complex shapes. Indeed, the method allows significant differences owing to composition, type of absorbing layer, and dimension of the dressing to be assessed, thus making it adaptable for the screening of multiple materials. In addition, it permits the testing of different volumes of exudate.

Regarding the behaviour of the different dressings, the results show that different dressings show different behaviour in terms of conformability, further confirming that this parameter is fundamental for evaluating dressing performance.

Regarding the measure of conformability, we can conclude that:

- There is a strong correlation between dressing composition and technology and its conformability. Instead, conformability is not affected by the amount of solution or by the size of the dressing but is characteristic of the dressing.
- While they show no impact on conformability, the use of different amounts of solution can impact absorption time in a manner that depends on the composition and size.
- Although capable of efficiently absorbing suitable volumes of liquid, dressings do not necessarily have good conformability.

In addition, significant differences are assessed in the time for absorption, the lateral spreading, and the ability to maintain a dry surface at the interface with the wound. All these parameters might impact the healing of the wound and should therefore be taken into consideration. At the same time, they do not necessarily correlate with conformability and should consequently be evaluated separately. For these reasons, to provide an accurate evaluation, the test should include absorption time and lateral spreading and consider different responses based on the exudate volume.

The proposed test represents a model for the assessment of conformability and, therefore, requires further clinical validation. However, it permits the determination of conformability, lateral spreading, capability to maintain a dry surface, and absorption time simultaneously, in a fast and easy way, without requiring complex equipment or designing tissue models. Hence, we believe it is promising for a fast and inexpensive determination of conformability of dressings. Future studies should demonstrate conformability in clinical settings and its impact on the healing of tissue (time and degree of tissue healing) and on the rise in infection. Indeed, different wound locations and types as well as the possible rise in infections might affect treatment of complicated wounds (such as those of very large dimensions), for example, by interfering with the adhesion of the dressing to the wound or its capability to seal the wound.

## Figures and Tables

**Table 1:** Amount of absorbed solution and absorption time.

Composition	Size	Liquid Volume	Weight Absorbed Liquid	β	Solution Absorption Time
Silicon foam	12 × 12	fence 30	7.27	0.97	instantaneous
Silicon foam	12 × 12	fence 45	16.72	1.00	instantaneous
Silicon foam	15 × 15	fence 45	16.54	0.99	instantaneous
Silicon foam	15 × 15	fence 60	29.59	0.99	instantaneous
Carboxymethylated spun cellulose fibres	12 × 12	fence 30	7.19	0.96	2–4 min
Carboxymethylated spun cellulose fibres	12 × 12	fence 45	16.5	0.98	4 min
Carboxymethylated spun cellulose fibres	15 × 15	fence 45	16.21	0.97	5 min
Carboxymethylated spun cellulose fibres	15 × 15	fence 60	29.99	1.00	7.5 min
Polyurethane foam	12 × 12	fence 30	7.3	0.98	instantaneous
Polyurethane foam	12 × 12	fence 45	16.37	0.98	instantaneous
Hydrocellular polyurethane foam	12 × 12	fence 30	7.15	0.96	2–4 min
Hydrocellular polyurethane foam	12 × 12	fence 45	16.35	0.97	<5 min
Hydrocellular lipido-colloid	12 × 12	fence 45	16.44	0.98	2–4 min
Hydrocellular lipido-colloid	12 × 12	fence 60	29.64	0.99	6 min

**Table 2:** Conformability measures.

Composition	Size	Liquid Volume	Visual Observation	Hnet,μ Average	St Dev	Alpha Ratio α	Notes
Silicon foam	12 × 12	fence 30	Bubble formation	14.12	2.12	0.47	0.48 [20]
Silicon foam	12 × 12	fence 45	Bubble formation	20.80	0.41	0.46	
Silicon foam	15 × 15	fence 45	Bubble formation	17.59	1.38	0.39	0.36 [20]
Silicon foam	15 × 15	fence 60	Bubble formation	19.98	1.26	0.33	
Carboxymethylated spun cellulose fibres	12 × 12	fence 30	Shape modification	0.53	0.84	0.02	<0.2 (LSL)
Carboxymethylated spun cellulose fibres	12 × 12	fence 45	Shape modification	0.09	0.53	0.00	<0.2
Carboxymethylated spun cellulose fibres	15 × 15	fence 45	Shape modification	1.28	0.90	0.03	<0.2
Carboxymethylated spun cellulose fibres	15 × 15	fence 60	Shape modification	1.81	0.29	0.03	<0.2
Polyurethane foam	12 × 12	fence 30	Minor shape modification (bending)	0.85	0.42	0.03	<0.2
Polyurethane foam	12 × 12	fence 45	Minor shape modification (bending)	2.65	0.68	0.06	<0.2
Hydrocellular polyurethane foam	12 × 12	fence 30	External layer detachment	2.36	1.54	0.08	<0.2
Hydrocellular polyurethane foam	12 × 12	fence 45	External layer detachment	1.69	0.73	0.04	<0.2
Hydrocellular lipido-colloid	12 × 12	fence 45	No modifications	0.59	0.13	0.01	<0.2
Hydrocellular lipido-colloid	12 × 12	fence 60	No modifications	0.00	/	0.00	<0.2

## Data Availability

The original contributions presented in this study are included in the article/Supplementary Materials. Further inquiries can be directed to the corresponding author (G.G.).

## Supplementary Materials

The following supporting information can be downloaded at: https://www.mdpi.com/article/10.3390/ijms27188149/s1.
