# Supplementary material for: New Facile Testing Protocols to Assess Conformability of Advanced Dressings for Wound Healing
Source: Int J Mol Sci. 2026 Sep 13;27(18):8149. doi: 10.3390/ijms27188149 (PMC13607526; doi:10.3390/ijms27188149)
Supplement: Supplementary file 1 [file ijms-27-08149-s001.zip › ijms-4510590-supplementary.pdf]

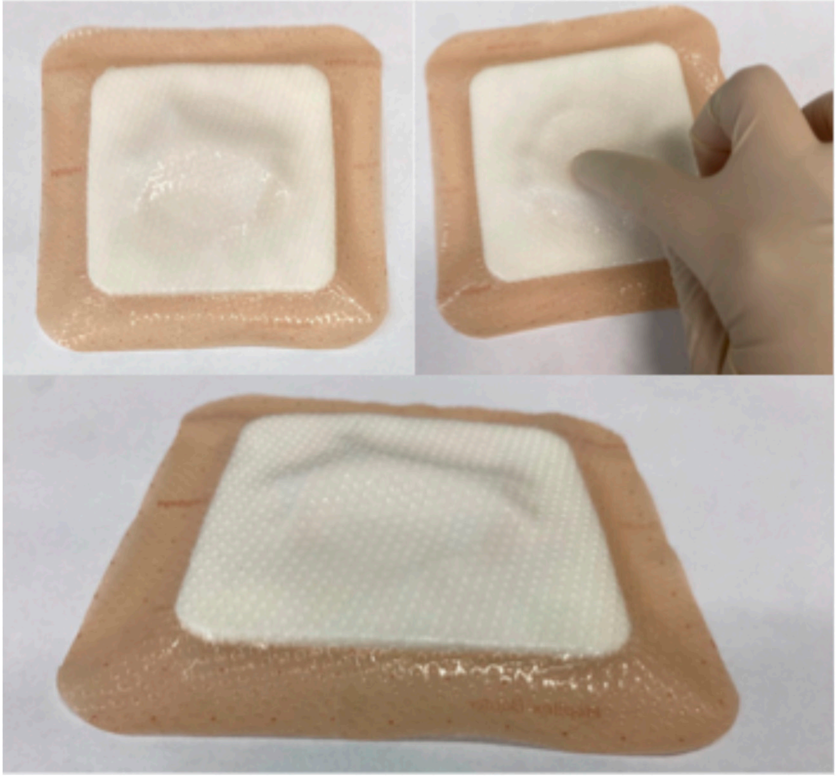

Figure S1: In the hydrocellular lipido-colloid dressing dressing a detachment of the upper layer is observed

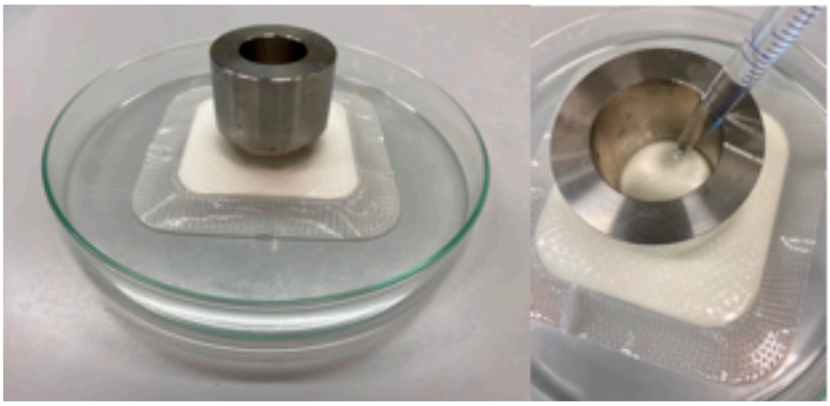

Figure S2: Testing procedure
